# Sphingosine Kinases promote IL-17 expression in human T lymphocytes

**DOI:** 10.1038/s41598-018-31666-1

**Published:** 2018-09-05

**Authors:** Giusi Barra, Alessio Lepore, Miriam Gagliardi, Domenico Somma, Maria Rosaria Matarazzo, Francesca Costabile, Giuseppe Pasquale, Alessio Mazzoni, Carmela Gallo, Genoveffa Nuzzo, Francesco Annunziato, Angelo Fontana, Antonio Leonardi, Raffaele De Palma

**Affiliations:** 1 Department of Precision Medicine, Università della Campania “L. Vanvitelli”, Napoli, Italy; 2 Institute of Genetics and Biophysics ‘Adriano Buzzati-Traverso’, CNR, Napoli, 80131 Italy; 3 Department of Experimental and Clinical Medicine and DENOTHE Center, University of Florence, Firenze, Italy; 4 Univeristy of Naples “Federico II”, Department of Molecular Medicine and Medical Biotechnology, Napoli, Italy; 5 Institute of Protein Biochemistry-CNR, via P. Castellino, 111, 80131 Napoli, Italy; 6 Bio-Organic Chemistry Unit, Institute of Biomolecular Chemistry-CNR, Via Campi Flegrei 34, Pozzuoli, 80078 Italy

## Abstract

Sphingosine 1-phosphate (S1P) has a role in many cellular processes. S1P is involved in cell growth and apoptosis, regulation of cell trafficking, production of cytokines and chemokines. The kinases SphK1 and SphK2 (SphKs) phosphorilate Sphingosine (Sph) to S1P and several phosphatases revert S1P to sphingosine, thus assuring a balanced pool that can be depleted by a Sphingosine lyase in hexadecenal compounds and aldehydes. There are evidences that SphK1 and 2 may per se control cellular processes. Here, we report that Sph kinases regulate IL-17 expression in human T cells. SphKs inhibition impairs the production of IL-17, while their overexpression up-regulates expression of the cytokine through acetylation of IL-17 promoter. SphKs were up-regulated also in PBMCs of patients affected by IL-17 related diseases. Thus, S1P/S1P kinases axis is a mechanism likely to promote IL-17 expression in human T cells, representing a possible therapeutic target in human inflammatory diseases.

## Introduction

Sphingosine 1-phosphate (S1P) is a phospholipid that mediates many signaling events and has a central role in several cellular processes^1,2^: it is essential for embryogenesis, cell trafficking, cell survival and apoptosis and plays many roles in immunity, inflammation and cancer^3–5^. S1P derives from Sphingomyelin, a lipid commonly found in cell membrane lipid rafts; Sphingomyelin is converted by sphingomyelinase into Ceramide, and finally is metabolized to Sphingosine (Sph) by ceramidase. Sphingosine, upon phosphorylation by two kinases, SphK1 and SphK2 is converted into S1P^1,2,5^. S1P can also be dephosphorylated by several phosphatases, or completely degraded by S1P lyase to phosphoethanolamine and hexadecenal compounds^1,2,5^. Intracellular levels of S1P are balanced by the equilibrium between its continuous formation and degradation, functioning as a rheostat that regulates different cellular processes, like cell growth and survival.

The relevance of S1P as a key molecule in the immune system has been growing in the last ten years, showing its crucial role in T and B cell chemotaxis, lymph node organization, mast cell and eosinophil functions and DC trafficking^1–8^. S1P gradient between periphery and lymph nodes is in fact critical for lymphocyte trafficking^6^, determining the egress of T and B lymphocytes and cell positioning in the nodes and spleen^6,7^.

The effects of S1P are mainly mediated by its binding to five G-protein coupled receptors (S1PR1-S1PR5)^1–5^. However, S1P does not act only through the binding of its receptors, but it may also act independently of its surface receptors^2,9^. For example, it has been shown that S1P may modulate HDAC1 and HDAC2 activity, therefore influencing gene expression directly^2,9,10^. Recently, several data suggest that Sph kinases, SphK1 and Sphk2, can account for several intracellular effect of S1P^9,11,12^, thus suggesting that the intracellular levels of S1P are important for its biological function also through Sph kinases mediated effects.

Indeed, the expression of SphK1 and SphK2 affects cell functions^4,5,13,14^. SphK1 has been described to be present mainly in the cytosol while SphK2 is mainly located in the nucleus^9–11^. Beside the phosphorylation of Sphingosine, these two kinases have many other functions that are far to be elucidated. We, and others, have shown that S1P/S1P kinases axis is crucial in bronchial hyperresponsiveness in allergic asthma^8,13,14^ and that this axis may affect cytokines production *in vitro* and in an animal model of disease^3,5,12–14^.

Here, we show that SphK1 and SphK2 play a role in the expression of IL-17, a cytokine mainly produced by Th17 lymphocytes, primarily involved in the defense against extracellular bacteria, fungi and protozoan infection^15,16^. Th17 cells sustain chronic inflammation and are of fundamental importance in autoimmune diseases; they are also expanded in many chronic inflammatory diseases, as Spondyloarthritis, Rheumatoid Arthritis, Psoriasis, Crohn Disease, Multiple Sclerosis, and in cancer although their role is still a matter of debate^17–21^.

In previous experiments of gene expression conducted on various human T cell subsets, we found several differences in the expression levels of genes accounting for S1P formation and metabolism^19,20^. In order to investigate the role of S1P and SphKs in regulating CD4 differentiation, here we analyze the expression of IL-17 in human T cell clones and human peripheral CD4+ T cells cultured in different polarizing conditions. Taking advantage of the availability of SphK1 and SphK2 inhibitors, and over-expressing the two kinases, we find that this axis promotes or impairs the expression of IL-17 in human T cells. Furthermore, we also studied the levels of expression of SphK1 and SphK2 in PBMCs of patients affected by spondyloarthritis, a disease typically associated to Th17 subset^20^, observing a correlation between the percentage of IL-17 producing cells, identified as CD4+/CD161+ double positive cells^19,20^, and the expression levels of SphKs.

Taken together, these data indicate that S1P/S1P kinases may represent a key target to modulate T cell functions specifically in conditions like chronic inflammatory diseases linked to IL-17 production.

## Results

### Human Th17 clones and peripheral polarized Th17 lymphocytes express higher SphKs mRNA levels

**Figure 1:**
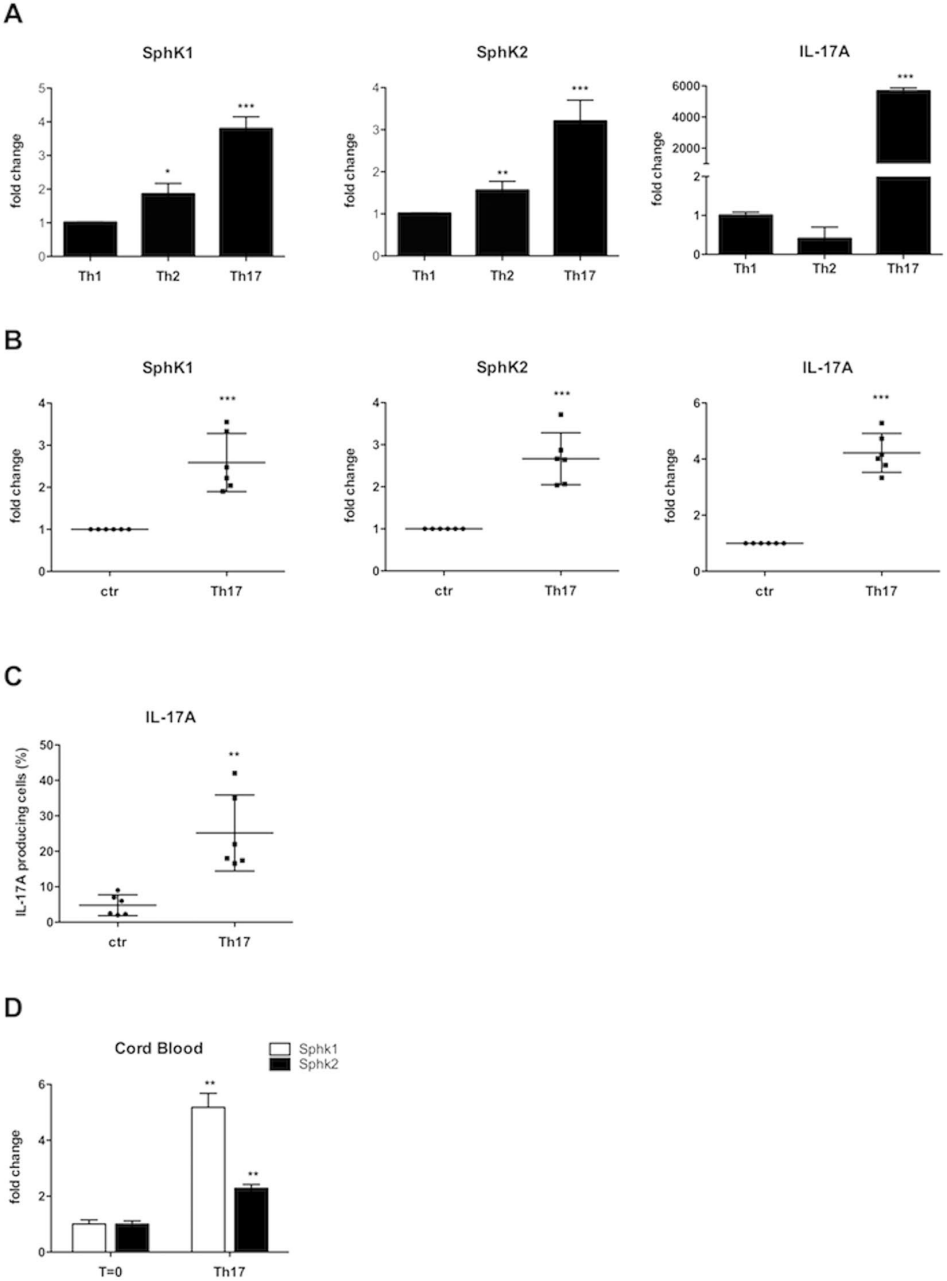
(**A**) mRNA Expression of SphK1 and SphK2 and IL-17A in human Th1, Th2 and Th17 clones. SphK1, SphK2 and IL-17A mRNA expression was evaluated by quantitative PCR. Results were normalized to 18S mRNA and analyzed by ΔΔCt method. Values on y-axis represent fold change in mRNA levels compared to control. Data indicate mean ± s.d. obtained from three separate experiments performed in triplicate. One-way ANOVA test followed by Tukey’s test were used for statistical analysis. *p < 0,01; ** < 0,001; ***p < 0.0001. (**B**) mRNA expression of SphK1, SphK2 and IL-17A in Th17 lymphocytes polarized *in vitro*. Peripheral TCD4+ cells cultured in Th17 polarizing condition; after12 days of culture, SphKs and IL-17A expression levels of CD4+ T cells (ctr) and polarized Th17 cells were evaluated by quantitative PCR. Results were generated as in Panel A. Values on y-axis represent fold change in mRNA levels compared to control. Data indicate mean ± s.d. obtained from six separate experiments performed in triplicate. Unpaired Two-tailed t-test was used for statistical analysis (***p < 0,0001). (**C**) IL-17A production in Th17 lymphocytes polarized *in vitro*. Graphic representation of IL-17A production evaluated by flow cytometry analysis. Unstimulated CD4+ T cells (ctr) and *in vitro* polarized Th17 were considered. After 12 days of cultures and a stimulation of 6 hours with PMA/ionomycin and BFA, cells were stained with anti human-IL-17A-Pe antibody for FACS analysis. Values on y-axis represent the percentage of IL-17A producing cells; The bars represent mean values ± s.d. obtained from six experiments. Unpaired Two-tailed t-test was used for statistical analysis (**p < 0,001). (**D**) SphKs mRNA expression by Cord blood CD4+ naive T cells. SphK1 and SphK2 mRNA expression by cord blood (CB) T cells was evaluated by quantitative PCR, at time 0 (T = 0) and after 6 days of culture with anti CD3/28 beads, IL-1, IL-23 and TGFβ (Th17). Results were generated as in Panel A Values on y-axis represent the relative gene expression in mRNA levels. Data indicate mean ± s.d. obtained from 3 separate experiments performed in duplicate. Unpaired Two-tailed t-test was used for statistical analysis: **p < 0,001.

To further exploit what we found in previous experiments on S1P/S1P kinases axis by gene expression array performed in human Th17 clones^19,20^, we compared the expression levels of SphK1 and SphK2 in human Th1, Th2 and Th17 clones by quantitative Real Time PCR analysis; as shown in Fig. 1A, both kinases were strongly up-regulated only in Th17 clones. Besides, we analyzed the protein levels of SphK1 and 2 by Western blot in Th17 compared to CD4+ T cells and, also in this experimental setting, the levels of both kinases were found significantly up-regulated (Supplementary Fig. S1, Panel A) in Th17 cells.

Once assessed the higher expression of SphKs in Th17 cells, we tried to check if it was a peculiarity of established T cell clones or it could be observed in a more “physiological” setting, that is during T helper cell polarization. To address this issue, we studied the SphKs expression in peripheral CD4+ T lymphocytes taken in culture in Th17 polarizing condition using a known specific mix of cytokines^19,20,22^. Polarizing conditions and the ability to acquire a Th17 profile were assessed monitoring the expression levels of different cytokines and transcription factor typically related to Th17 profile, STAT3, RORC2, IL-17F and IL-22 (Supplementary Fig. S1, Panel B). In these experiments, after twelve days of culture we analyzed the expression levels of SphK1, SphK2 and IL-17A, and we found that CD4+ T helper cells showed an increase of SphK1 and SphK2 mRNA, indicating that the augmented expression of the two SphKs is paralleling the ability to produce IL-17 (Fig. 1, Panel B) as proven by studying IL-17 mRNA, and also intracellular production of IL-17 (Fig. 1, Panel C). Experiments were conducted independently on six different buffy coats obtained from healthy donors. Different results were obtained when peripheral T CD4+ cells were cultured in Th1 or Th2 polarizing conditions and, in both subsets, SphK1 expression was lower than the one observed in non polarized TCD4+ cells. No significant differences were found for SphK2 (Supplementary Fig. S1, Panel C).

We observed an increased expression of SphKs both in Th17 clones and in peripheral CD4 T cells grown in Th17 polarizing conditions. To rule out if this phenomenon was merely due to expansion of Th17 memory cells or it had a role in the acquisition of the ability to produce IL-17, we decided to investigate the expression levels of SphK1 and SphK2 also in naïve CD4+ T cells. To this aim, we used human Umbilical Cord Blood (UCB) and we studied the expression of the two kinases in UCB cells, cultured in Th17 polarizing condition, according to a known protocol^19,20^. Figure 1, Panel D shows that SphK1 and SphK2 mRNA expression levels were increased also in these cells after one week of culture, thus suggesting that the increased expression of SphK1 and SphK2 was going along with the ability to produce IL-17.

### Inhibition of SphK1 and SphK2 impaired the ability of CD4+ T helper lymphocytes to produce IL-17

Taken together, the data obtained so far demonstrate that SphK1 and SphK2 associate with the ability to express IL-17 and become Th17 cells, suggesting that these kinases may play a role in the production of IL-17.

**Figure 2:**
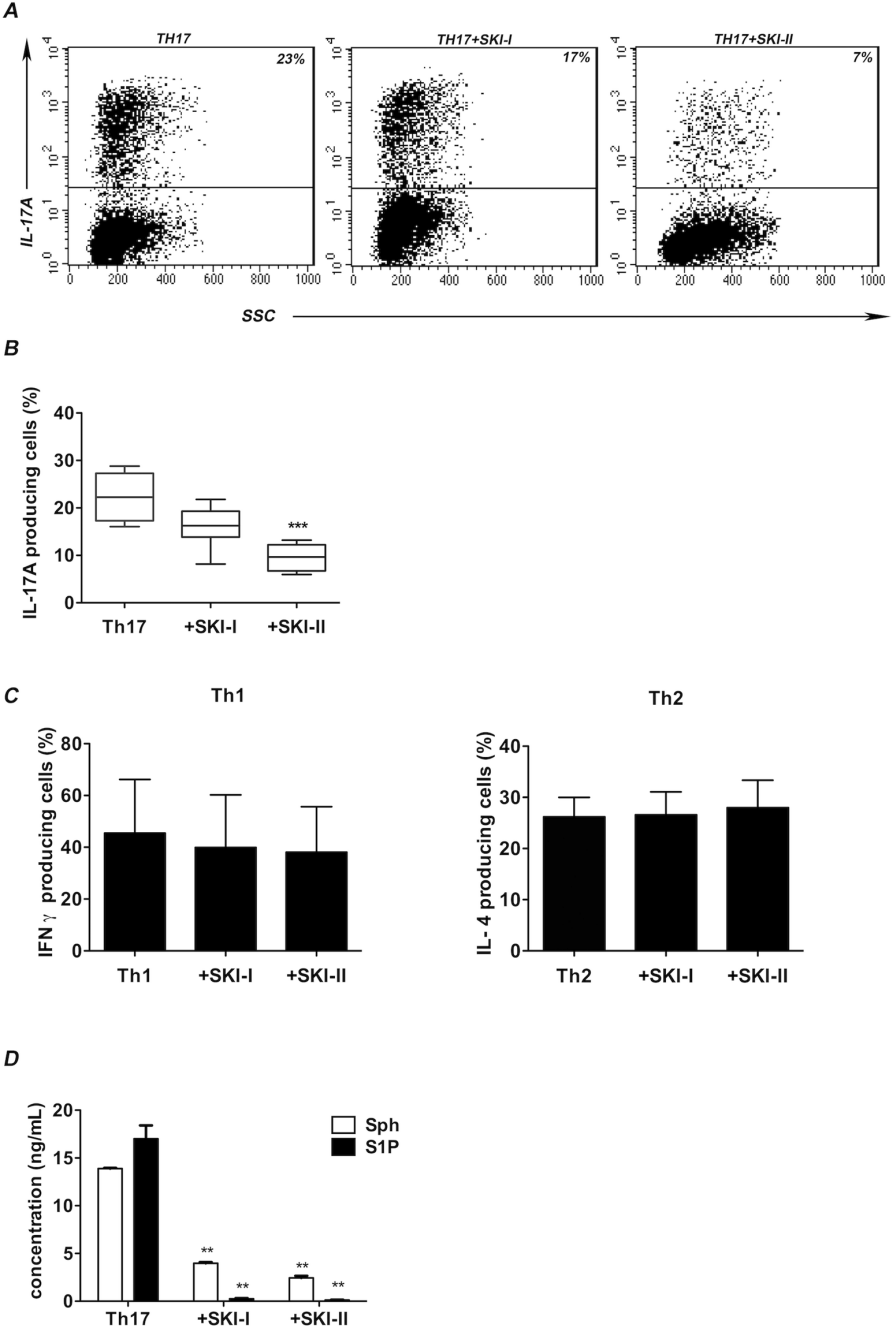
(**A**) IL-17A production is reduced after SKIs treatment. Representative flow cytometric analysis of IL-17A production by Th17 polarized cells, compared to polarized Th17 cells cultured in the presence of SKI-I or SKI-II after stimulation with PMA plus ionomycin and BFA for 6 hours. One representative experiment out of the six performed is shown. Numbers in plots indicate percentage of gated cells. The gates were placed on the basis of an isotype-matched control mAb. (**B**) SKIs effect on Th17 lymphocytes graphic representation of the flow cytometric analysis conducted on all the six experiments. Graph represents the reduced production of IL-17A after the treatments with SKI-I (Th17 + SKI-I) or SKI-II (Th17 + SKI-II) inhibitors, compared to cells polarized without inhibitors (Th17). The bars represents mean values ± s.d. Values on y-axis represent the percentage of IL-17A producing cells; One way ANOVA test, followed by Tukey’s test were used for statistical analysis. ***p < 0,0001. (**C**) SKIs effect on Th1 and Th2 lymphocytes. Graphic representation of IFN-γ production in Th1 and IL-4 in Th2 cells when polarized *in vitro* with or without the treatments with SKI-I or SKI-II inhibitors; cells polarized without inhibitors (Th1 or Th2) were used as control. Flow cytometry analysis was conducted after 12 days of culture in presence of IL-12 or IL-4, and after a treatment of 6 hours with PMA/Ionomycin and BFA. Values on y-axis represent the percentage of IFN-γ or IL-4 producing cells. The bars represent mean values ± standard deviation obtained from six experiments. One-way ANOVA test, followed by Tukey’s test were used for statistical analysis. (**D**) Sph and S1P production in TCD4+ supernatants after SphKs inhibition. Level (ng/mL) of S1P and Sph were tested in supernatants of CD4^+^ and Th17 T cells in the presence or absence of inhibitors SKI-1 and SKI-II, after 12 days of culture. The internal standards (C17-Sph and C17-S1P) were added to the supernatants, which were processed by HRX-SPE (Chromabond®); The recovered fractions enriched in S1P and Sph were analyzed by LC-MS-MSMS. Bars indicate mean ± s.d. One-way ANOVA test, followed by Tukey’s test were used for statistical analysis. (**p < 0,01).

To test this hypothesis, we performed Th17 cells polarization experiments in the absence or in the presence of two chemical compounds, SKI-I and SKI-II, known to block SphK1 and both SphK1 and SphK2 respectively^23^. Figure 2, Panel A shows a representative experiment demonstrating that SKI-I and SKI-II induce a significant reduction of the frequency of IL-17A producing cells when added to the cultures. Figure 2, panel B, summarizes data of the same experiment performed on six different buffy coats. Of note, SKI-II blocking both SphK1 and SphK2, reduces the frequency of IL-17A producing cells clearly more than SKI-I alone. Conducting the same experiments in Th1 or Th2 polarizing condition, we did not find significant variation in IFNγ or IL-4 production, respectively (shown in Fig. 2 panel C), thus further suggesting a correlation between SphKs activity and Th17 differentiation.

### Determination of S1P and Sphingosine levels by inhibition and overexpression of SphK1 and SphK2

To test the functional effects of the inhibitors used, we performed some experiments to measure the amount of Sphingosine (Sph) and S1P in the supernatants of Th17 polarized *in vitro* with or without SKIs through HPLC-MS technique, as detailed in Material & Method section. Figure 2, Panel D shows that the inhibition of SphKs by SKI-I and SKI-II lowered S1P and Sphingosine in Th17 cells. In the treated cells, the levels of both substances underwent a drastic reduction in comparison to control. However, S1P values were more affected and fell almost below the threshold of detention, indicating that the use of inhibitors was impairing the S1P/S1P kinases axis mainly affecting S1P formation as expected.

### Inhibition of SphK1 and SphK2 does not affect expression of master genes regulating Th17 polarization

**Figure 3:**
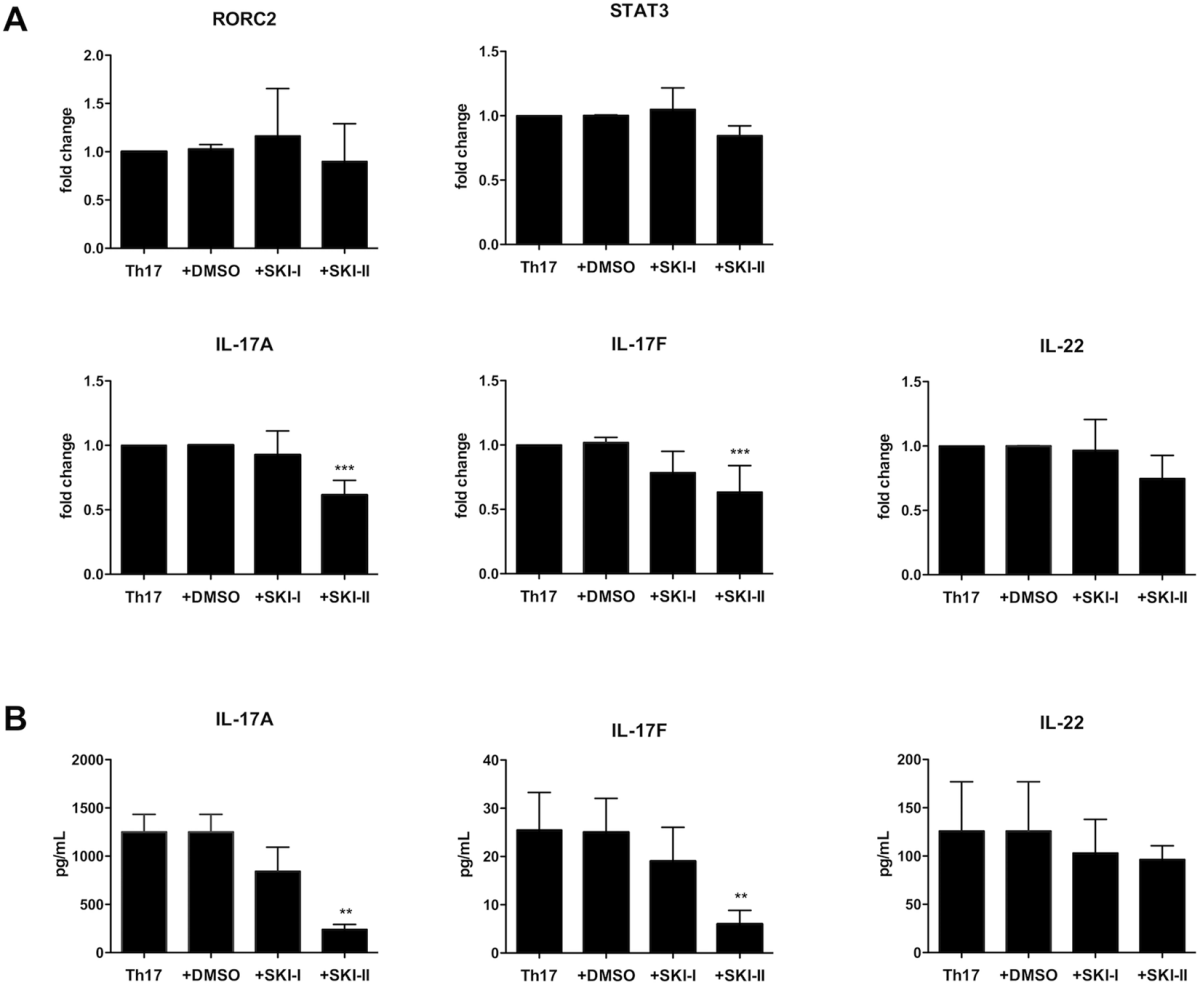
(**A**) mRNA expression of Th17 markers after the treatments with inhibitors. RORC2, STAT3, IL-17A, IL-17F and IL-22 mRNA expression by Th17 polarized cells (Th17) compared to Th17 polarized cells, cultured in the presence of the vehicle DMSO (Th17 + DMSO), of SKI-I (Th17 + SKI-I) or SKI-II (Th17 + SKI-II) was evaluated quantitative PCR. Results were normalized to 18 S mRNA and analyzed by ΔΔCt method. Values on y-axis represent fold change in mRNA levels compared to control. Data indicate mean ± s.d. obtained from six separate experiments performed in triplicate. One way ANOVA test, followed by Tukey’s test were used for statistical analysis. *p < 0,05; **p < 0,01; ***p < 0,001. (**B**) Cytokines production in supernatants of Th17 polarized cells. IL-17A, IL-17F and IL-22 production in supernatants of polarized Th17 cells (Th17), polarized Th17 cells cultured in the presence of vehicle DMSO (Th17 + DMSO), inhibitor SKI-I (Th17 + SKI-I) or with SKI-II (Th17 + SKI-II). Supernatants were collected at day 12 of culture. Cytokines production was measured with human multiplex-cytokine kits (Millipore) using Luminex technology. Bars indicate mean ± s.d. obtained from four separate experiments performed in duplicate. One way ANOVA test, followed by Tukey’s test were used for statistical analysis. **p < 0,01.

Next, we wanted to address if the activity of SphKs was important for Th17 polarization status rather than for the production of IL-17. To this aim, using the same experimental conditions we analyzed the expression of RORC2 and STAT3 two transcription factors characterizing Th17 polarization, and production of IL-17A, IL-17F and IL-22, cytokines that are hallmarks of Th17 profile. Figure 3, Panel A, shows that the use of SphKs inhibitors significantly inhibits the message for IL-17A and IL-17F. On the opposite, the expression of RORC2 and IL-22 are not affected, suggesting that the kinases activity is mainly involved in IL-17 production. Of note we detect also slight decrease of STAT3 mRNA, that could possibly further contribute to the decreased expression of IL-17. The impairment of IL-17A and IL-17F production was also confirmed measuring the proteins in the cell supernatant by Luminex assay. Similarly, we did not find significant variation in IL-22 protein, as found for analysis of the corrisponding gene expression (Fig. 3, Panel B). These results suggest that SphKs may play a role in determining the ability to produce IL-17 and may eventually regulate STAT3 expression. With these experiments we observed again that the inhibition of both kinases using SKI-II was more effective than the block of the only SphK1 with SKI-I, suggesting that both kinases are critical for IL-17 production. However, we could not exclude the possibility that these results may be due to mechanism other than kinases inhibition upon use of SphKs inhibitors in culture.

### Overexpression of SphK1 and SphK2 increases expression of IL-17

To better characterize the specificity of these two enzymes in inducing IL-17 production, we decided to study the functional effects of SphK1 and SphK2 ectopic expression in T helper.

We transiently transfected peripheral T CD4+ lymphocytes with SphK1 or SphK2 using the nucleofection technique; as pharmacological inhibition revealed that both kinases were useful for IL-17 expression, we also transfected cells with the two kinases together.

Only experiments associated to a transfection efficiency of live cells higher then 40% were considered; Sphks expression was also verified through western blot assay (Supplementary Fig. S2, Panel A and Panel B).

**Figure 4:**
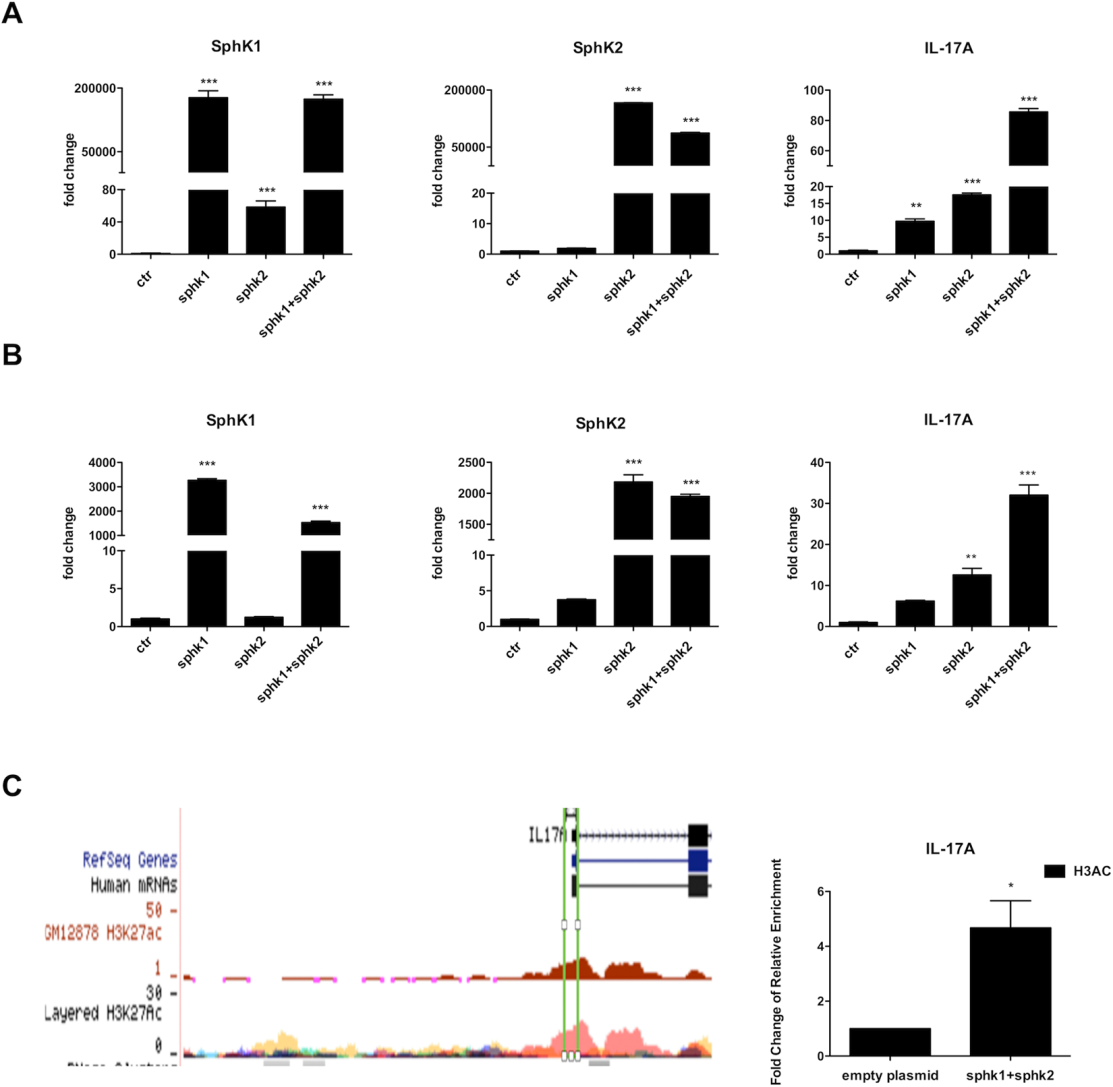
SphKs transfection. SphK1 and SpK2 and IL-17A mRNA expression levels were evaluated for: (**A**) CD4+ T cells and (**B**) for Jurkat T cell line, after nucleofection (Amaxa nucleofector technology-Lonza) with 2 μg of vectors containing SphK1, SphK2 or SphK1 + SphK2 overexpressed; empty plasmid was used as control. After 48 hours of culture, mRNA expression was evaluated by quantitative PCR. One representative experiment is shown. Results were normalized to 18 S mRNA and analyzed by ΔΔCt method. Values on y-axis represent fold change in mRNA levels compared to control. One way ANOVA test followed by Tukey’s test were used for statistical analysis.: **p < 0,001; ***p < 0,0001. (**C**) SphK1 and SphK2 overexpression induces significant increase of H3 Acetylation at activated IL-17 gene. The graph on the right represents a ChIP Assay followed by qPCR. It shows increased H3 Acetylation (H3Ac) at IL-17 transcriptional start sites. Results were normalized to the IgG (negative control) and compared with the enrichment in the control sample (empty plasmid). One representative experiment is shown. Values on y-axis represent the fold change of relative enrichment compared to the negative control. Data indicate mean ± s.d. Unpaired Two-tailed t-test was used for statistical analysis. *p < 0,05. The left panel represents the position of the region analyzed with the acetylation profiles of H3 in different cell lines.

Figure 4, Panel A, shows a significant up-regulation of IL-17A message in T cells transfected with both kinases. Luminex assay conducted after 48 hours on supernatants of these cells cultures, confirmed that IL-17A and IL-17F but not IL-22 protein production is increased upon kinases transfection (Supplementary Fig. S3, Panel A).

It may be argued that peripheral T cells are most likely memory cells, thus having a cell program already prone to make IL-17. Therefore, to better understand the role of SphK1 and SphK2 in IL-17 production, we performed the same experiments using a Jurkat T cell line that do not produce IL-17. Also in this case we observed a massive fold increase (about 40) for IL-17 message (Fig. 4, Panel B) further suggesting that SphKs plays a critical role in IL-17 production.

As we did for inhibition experiments, also in these experiments we measured the levels of Sph and S1P in cell cultures supernatants. We observed that the overexpression of the two kinases dramatically increase S1P (Supplementary Fig. S3, Panel B), thus confirming the efficiency of our model.

### SphK1 and SphK2 overexpression induces increase of H3 Acetylation at IL-17 promoter

It has been previously shown that S1P/SphKs specifically bind to the histone deacetylases HDAC1 and HDAC2 and inhibit their enzymatic activity, preventing the removal of acetyl groups from lysine residues within histone tails. SphK2 associate with HDAC1 and HDAC2 in repressor complexes and is selectively enriched at the promoters of specific genes, where it enhances local histone H3 acetylation (H3Ac) and transcription^9^. Thus, HDACs are direct intracellular targets of S1P and link nuclear S1P to epigenetic regulation of gene expression. To provide evidence that up-regulation of IL-17A was mediated by changes of H3Ac upon SphK1 and SphK2 overexpression, we analyzed this histone mark by Chromatin Immunoprecipitation assay. CD4+ T cells overexpressing both kinases showed increased level of H3Ac at IL-17A promoter (Fig. 4, Panel C), confirming that SphK1 and SphK2 regulate the activity of HDACs at the promoter of IL-17 gene.

### Increased expression of SphKs in peripheral CD4+/CD161+ T cells of patients affected by Th17 related disease

**Figure 5:**
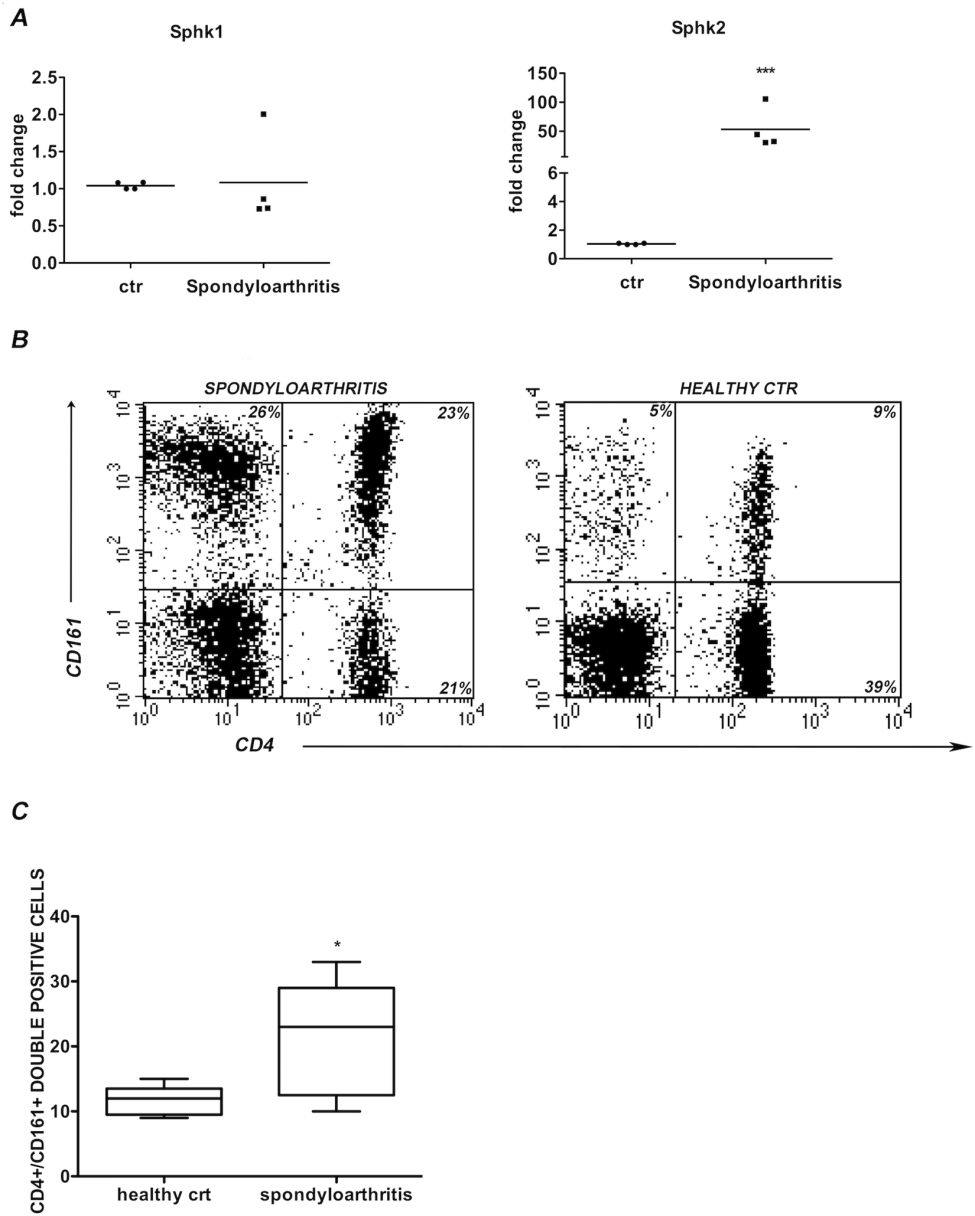
(**A**) SphKs mRNA expression in peripheral blood samples of patients affected by spondyloarthritis. PBMCs were isolated with ficoll from peripheral blood of patients affected by spondyloarthritis and from healthy controls (ctr). mRNA was extracted and reverse transcripted into cDNA. SphKs expression levels were evaluated by Real time qPCR. Results were normalized to 18 S mRNA and analyzed by ΔΔCt method. Values on y-axis represent fold change in mRNA levels compared to control. Data indicate mean ± d.s. obtained from four different samples. One-way ANOVA test followed by Tukey’s test were used for statistical analysis. ***p < 0.0001. (**B**) CD4+/CD161+ T cells in spondyloarthritis patient and healthy controls. Graphic representation of CD4+/CD161+, in PBMCs sample of a patient affected by spondyloarthritis (left plot) compared to an healthy control (right plot). One representative experiment is shown. Flow cytometric analysis was conducted after a staining with anti CD3, CD4, CD161 antibodies. Numbers in plots indicate percentage of gated cells. The gates were placed on the CD3+ population; 10.000 CD3+ cells were acquired. The up-right quadrant represents the population double positive for CD4 and CD161 (23% or 9%).

We observed a tight correlation between SphKs expression and IL-17 production, so we wanted to investigate if this link could be found also in peripheral blood of patients affected by immune-mediated diseases known to associate with Th17 responses. The prototype of Th17-related disease is spondyloarthritis, the human disease in which the Th17 were first identified^21^. We then analyzed peripheral blood samples obtained from patients affected by spondyloarthritis. To this aim, we performed a quantitative Real Time PCR analysis that revealed that the PBMCs of these patients had higher expression of SphKs, in particular SphK2, when compared to healthy controls (Fig. 5, Panel A). Moreover, by FACS analysis we measured the percentage of peripheral CD3+ T cells double positive for CD4/CD161, a specific phenotype associated with Th17. Figure 5, Panel B shows one representative experiment where it is evident the increase of Th17 cells in patients when compared to healthy control. As shown in Fig. 5, Panel C, this trend is visible in all the five samples analyzed.

### S1P levels are increased in patients having spondyloarthritis

**Figure 6:**
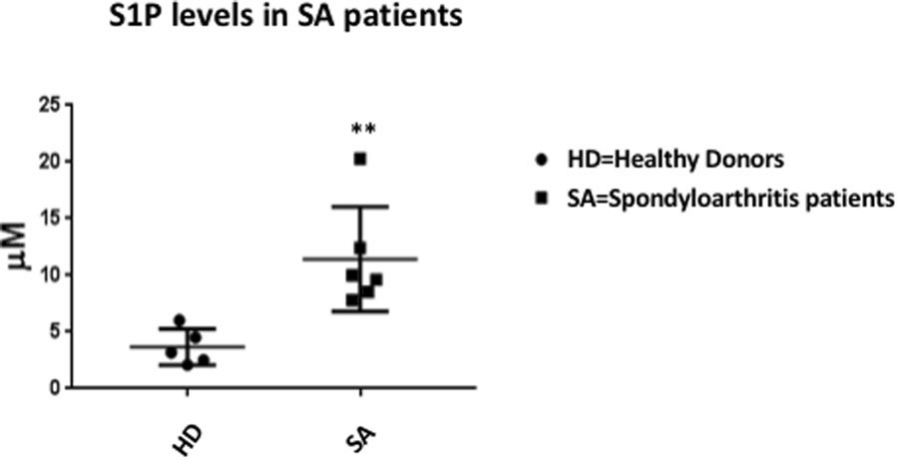
S1P levels in patients affected by spondyloarthritis (SA) and healthy controls. Measurement of S1P levels in 6 patients (■) affected by spondyloarthritis compared with S1P levels measured in 5 Healthy Donors (•). S1P levels were significantly higher in SA patients (**p < 0.005 by Mann-Whitney test).

Finally, we measured S1P levels in sera of patients affected by spondyloarthritis (SA), and we found that S1P levels in SA patients were significantly higher, suggesting that increased kinases activity was functionally related to an increase of S1P in a “Real Life” conditions, in a Th17 (Fig. 6).

## Discussion

CD4+ T helper cells play a pivotal role in immune system functioning through the production of a wide array of cytokines^18^. The ability to produce a certain set of cytokines led to the identification of T cell subsets having importance in different types of immune responses. Among T helper subsets, Th17 have a main role in protecting against extracellular bacteria and fungi. The other side of the immune responses is autoimmunity and it has been shown that Th17 have a peculiar role also in chronic inflammatory diseases and autoimmunity, as well as in cancer^15,16^. It has been reported that S1P may have an important role in orchestrating immune responses in many different ways. It has been suggested that S1P can also be involved in Th17 differentiation and may act per se on T cell polarization^18,24^.

Besides S1P ability to regulate cell trafficking, especially recruitment of T cell from lymph nodes, there are increasing evidences that S1P may directly interfere with the production of chemokines and cytokines. In this aspect, there have been many evidences that the two Sphingosine kinases that regulate S1P formation may directly be involved in regulation of cellular processes and, eventually, contributing to production of cytokines by different cells^8,13–16^.

Goetzly *et al*., in 2008 focused on S1P/S1P receptor 1 axis and found a clear association with the terminal differentiation of Th17, proposing an equal activity for S1P and IL-23^18,24^. More recently, several papers reported a link between S1P/S1P kinases axis and Th17^25,26^. Here we report that turning on and off Shingosine kinases has a direct effect on IL-17 production. We started showing that the SphKs are selectively over-expressed in human Th17 clones. Then, we demonstrate that inhibition of the two SphKs impair production of IL-17 by human peripheral CD4+ T cells cultured in Th17-polarizing conditions, implying a role for the kinases in the production of the cytokine during the polarization process. To further understand the meaning of these findings, we characterized the polarization status of T helper cells in these experiments and we found that the Sphingosine kinases axis has a role on IL-17 production more than on Th17 polarization itself. Indeed, the blocking of SphKs impaired IL-17 production while it did not affect the expression of RORC2, a master gene for Th17 differentiation, and did not affect production of IL-22, a cytokine associated to Th17 polarization status. Moreover, overexpression of SphKs is linked to production of IL-17 also in naïve cells, as shown by the experiments performed using cord blood T cells. Finally, over-expression of SphKs promotes secretion of IL-17 also in Jurkat T cells that do not produce IL-17 normally. Taken together, these experiments suggest that the Sphingosine kinases are involved in the regulation of IL-17 production although they do not affect the transcription factors regulating Th17 development as RORC2. Of interest, the impairment of Shingosine kinases had also a slight effect on STAT3, suggesting that this axis may be critical to control essential processes in chronic inflammation and cancer through STAT3. This is in line with previously reported data showing that the persistent activation of STAT3, upon SphK1 up-regulation, led to colitis-associated cancer in mouse model, linking S1P to chronic inflammation and cancer through STAT3 activation^27^ and, at least in part, the cancer development seems in these experiments mediated by promoting Th17 cells^28^.

We also performed several experiments aiming to silence SphKs, we found that double silencing for both kinases was inducing cell death extensively, while the silencing of the single kinase had a variable efficiency (*data not shown*), and we found similar results in our experiments when we inhibited a single SphK. This is not surprising in light of the pleiotropic functions of the two kinases and further stress the importance of this axis.

Data discussed so far demonstrated that over-expression of the two SphKs induce production of IL-17 in human T helper cells, linking directly S1P/S1P kinases axis to IL-17 production. The successive step was the attempt to better define how the kinases could induce the cytokine production, and we found that the effects of SphKs are mediated by acetylation at IL-17 promoter site upon over-expression of SphKs, thus suggesting that this entire axis might be crucial in reprogramming cell functions epigenetically after stimuli. To this respect, the ability to induce gene transcription of cytokines by Sphingosine Kinases has been recently reported^9,29^. To date, the main known mechanism controlling SphKs activity is the alteration of S1P pool. This may be of physiological relevance in several circumstances. For instance, it has been shown that S1P lyase is produced by intracellular pathogens as Lysteria species and Mycobacterium species^30,31^. During infections S1P lyase produced by pathogens deplete S1P intracellular pool, compromising many cell pathways. Therefore, we may hypothesize that expression of SphKs aimed to counteract alterations of S1P pool is a conserved axis, probably evolutionary linked to resistance against infections. We will conduct future experiments to further clarify this issue as well as molecular activity of the two SphKs.

In this scenario, it could be possible that chronic perturbations of this axis, as ongoing exposure to some infections, may concur to promote IL-17 production and chronic inflammation underlying autoimmune diseases as well as other chronic inflammatory diseases (i.e. Atherosclerosis). Of note, we have previously observed that S1P lyase is profoundly down-regulated in human Th17 clones^19^, further suggesting that maintaining S1P pool avoiding sphingosine depletion may be a fundamental mechanism to promote IL-17 related functions by T helper. In our experiments, we observed an impaired Th17 polarization and a decrease in Sphingosine and S1P in the presence of inhibitors, further supporting the hypothesis that S1P pool and S1P/S1P kinases axis is critical for IL-17 production.

We also proved that overexpression of SphKs may be found in pathological conditions, in humans. In fact, we showed that overexpression of these kinases is observed in parallel with increased frequency of Th17 cells in patients affected by spondyloarthritis (SA), a conditions from long time known to be associated to IL-17. Here, we observed an increase of S1P levels in blood of SA patients when compared to healthy donors, thus further suggesting that activities of the SphKs was functionally leading to an increase in S1P. These data are in line also with recent studies conducted on samples of psoriatic patients which showed a clear correlation between the stage of disease and the increase of S1P production, associated to a reduction in ceramide production^32^.

In this line, recently it has been shown that SphKs/S1P axis is involved in SA at multiple levels^33^, indeed in SA patients this axis may promote IL-17 production and directly regulate metabolic pathways determining the mineralization capacity of osteoblasts and chondrocytes in SA, suggesting that this axis may be a peculiar therapeutic target in spondyloarthritis.

To this regard we want to stress that the modulation of Sphingosine kinases has been shown to be useful to treat animal model of autoimmune disease and in cancer^11,26,27,34,35^, probably affecting many different processes at multiple levels.

This is even more appealing since many chemical compounds able to act on SphK1 and SphK2 are available and many more are coming^32^.

Altogether, the experiments reported here suggest that S1P/S1P kinases axis may have a fundamental role in promoting IL-17 production more than Th17 polarization, acting through IL-17 promoter acetylation. As consequence, we may envisage a scenario in which alterations of S1P pool and perturbation of SphKs represent a signal leading to IL-17 production, and may be a key pathway originally developed to fight infections whose modulation could be of great interest in several pathological conditions linked to IL-17 production. These speculations may also lead to new hypotheses and explanations to better understand development of chronic inflammatory diseases related to IL-17 production.

In conclusion, we show here that S1P/Sphingosine kinases axis is critical for IL-17 production and may represent a key target to modulate IL-17 production, representing a possible therapeutic target in many different conditions. Further studies are needed to detail the effect of this axis, and the behavior and role of the single kinases on different cell functions.

## Materials and Methods

All human samples were obtained after a written informed consensus given by patients and healthy donors. The use of these samples for research purposes was approved by the Ethical Committee of the Dept. of Clinical and Experimental Medicine at the University of Campania “L. Vanvitelli”. All methods were performed in accordance with the relevant guidelines and regulations.

### Human T cell clones

Generation and characterization of human Th1, Th2 and Th17 clones and umbilical cord blood (UCB) cells isolation and polarization has been detailed previously^19,20^.

### Isolation and culture of human CD4+ T Lymphocytes

Peripheral blood mononuclear cells (PBMCs) from healthy donors were isolated by Ficoll-Paque Plus (GE Healthcare) and CD4+ T cells were separated by positive selection using human CD4 microbeads (Miltenyi biotec) and passed over LS columns to achieve purities >99%. CD4+ T cells obtained, were cultured for 6 days in 96 well U bottomed plates at a density of 1 × 10^5^ cells per well in complete medium (200 μL) composed by RPMI 1640 containing human AB serum (10%) (Sigma Aldrich), Ultraglutamine I (1%), penicillin and streptomycin (1%) along with beads coated with anti-CD3 and anti-CD28 (Life Technologies) at a ratio of 1 bead per 10 cells.

T cell culturing in polarizing conditions has been described as well^22^.

### Patients samples

Peripheral blood of patients affected by IL-17 correlated disease was drawn after their informed consent (see above). PBMCs were obtained after gradient stratification on Ficoll-Paque Plus (GE Healthcare); cells were stained with monoclonal antibodies anti CD3 Percp, CD4 Fitc, CD161 Pe (Miltenyi biotech) for FACS analysis; PBMCs mRNA was also extracted and reverse transcribed for qPCR Real Time analysis, as described below. Sera were obtained according to a standard protocol, by blood centrifugation, and S1P levels were measured as below described.

### Sphingosine Kinases Inhibitors

The effect of SphK1 and SphK2 inhibition on CD4+ T cells fate was assessed culturing 1 × 10^5^ cells/well in medium (200 μL), in polarizing condition as before, with or without pharmacological inhibitors SKI-I (5 μM; Tocris bioscience) or SKI-II (5 μM; Enzo life science), both resuspended in DMSO.

### Flow cytometry

After separation with microbeads, CD4+ T cells were stained with mouse anti-human monoclonal antibodies: CD14-Fitc or CD8-Fitc, CD4-Pe, CD3-PercP and the isotype control (Miltenyi Biotec) and analyzed by flow cytometry to assess purity.

The analysis of intracellular cytokine production was done after 12 days of culture; T cells were stimulated for 6 hours with phorbol 12-myristate 13-acetate (PMA, 10 ng/mL), Ionomycin (500 ng/mL) and Brefeldin A (BFA 10 μg/mL) (Sigma Aldrich) and the intracellular staining was performed incubating T cells with mouse monoclonal antibody IL-17Pe (Miltenyi Biotec), T cells were acquired on a FACScalibur (BD Biosciences) and analyzed with Cell Quest software (BD Biosciences).

### RNA extraction, cDNA reverse transcription and Quantitative Real Time PCR

T cells total RNA was extracted using Trizol reagent (Life Technologies). Reverse transcriptase reaction was carried out to convert the RNA isolated (1 μg) into cDNA using Super-Script reverse transcriptase III (Life Technologies) according to the manifacturer instruction.

Expression levels of genes encoding for: SphK1, SphK2, IL-17A, IL-17F, IL-22 and STAT3, RORC2 were analyzed using Real time quantitative PCR. Gene-specific primers are reported in Supplementary Table S1. Amplifications were done using the SYBR Green PCR Master Mix (Applied Biosystems). The thermal cycling conditions were composed of 50 °C for 2 min (stage 1) followed by a denaturation step at 95 °C for 10 min (stage 2) and then 40 cycles at 95 °C for 15 s and 60 °C for 1 min (stage 3). All samples were run in triplicate (25 μL), using a 7500 Real Time PCR system (Applied Biosystems) and relative expression of genes was determined by normalizing to 18S, used as internal control gene; to calculate relative gene expression in value it was used the 2^− ΔΔCt^ method. Nonspecific signals caused by primer dimers were excluded by dissociation curve analysis and use of non-template controls.

### T cell transfection

Jurkat T cell line and normal T CD4+ lymphocytes were transfected with pcDNA3 plasmid empty or containing SphK1 and SphK2 cDNA, using Amaxa cell line nucleofector kit V and human T cell nucleofector kit (Lonza) respectively, according to manufacturer instruction. Briefly, cells were resuspended in nucleofector solution (100 μl). plasmidic DNA (2 μg) were added and transfected using the suggested program. Cells were transferred to a prewarmed 12well plate containing RPMI with 10% human AB serum (2 mL) and incubated at 37 °C. Five hours later, medium was changed and for T cells were also added beads coated with anti-CD3 and anti-CD28 (Life Technologies) in a ratio of 1 bead per 10 cells. Cells were harvested 48 hours after nucleofection. Transfection efficiency was controlled with a green fluorescent protein (GFP)–expressing vector (Amaxa) by flow cytometry

### Western blot

After transfection, cells were washed with phosphate-buffered saline and lysed using a lysis buffer (50 mM Tris-HCL, 150 mM NaCl, 1% NP-40) containing protease and phosphatase inhibitors cocktail tablet. Equal amounts of protein were resolved by SDS–PAGE analysis using 10% polyacrylamide gels and transferred to a nitrocellulose membrane. Membranes were blocked with non-fat milk (5%) in Tris buffered saline (TBS, pH 7.4) containing 0.1% Tween-20 (TBS-T), for 1 h at room temperature. After rinsing, membranes were incubated with antibodies according to the manufacturer instruction against FLAG (Sigma Aldrich F7425), actin (Sigma Aldrich), SphK1 and SphK2 (Cell Signaling; Abcam). After three washing in TBS-T buffer, the nitrocellulose membranes were incubated with a HRP conjugated anti-rabbit IgG antibody (Sigma Aldrich). Detection was accomplished by chemiluminesence using ECL detection reagent and exposed to Hyperfilm-ECL film (GE Healthcare).

### Chromatin Immunoprecipitation Assay

Chromatin immunoprecipitation (ChIP) was performed according to a described protocol^35,36^. Briefly, 1 × 10^6^ cells were fixed with formaldehyde (0.3%), followed by glycine (125 mM) to stop the crossing linking reaction. Nuclear extracts were sonicated using the Covaris S2 system sonicator to achieve chromosome fragment lengths of 200–500 bp. Suitable amount of chromatin was then incubated with the H3Ac antibody (Abcam). Immunoprecipitated complexes were recovered using protein A sepharose and samples were then washed with low and high salt buffers, reverse-crosslinked, and purified using the QIAquick PCR purification kit (Qiagen). Purified DNA was analyzed by qRT-PCR using gene-specific primers (S1B)

### Sample preparation for LC/MS analysis

Standard C17-Sph and C17-S1P were prepared of both lipids (stock solutions of 500 µg/mL in MeOH). After addition of these standards (500 ng), cell supernatant (1 mL) were processed by Solid Phase Extraction (SPE) on CHROMABOND^®^ HR-X cartridges (6 mL/500 mg). The extraction was carried out on GX-271 ASPEC Gilson apparatus by a modified protocol of the method reported in literature^23^ Briefly, supernatants were directly loaded onto the pre-packed HR-X cartridges and submitted to a preliminary desalting step with distilled water (8 mL). Then, organic components were recovered from the column by elution with CH_3_CN/H_2_O 70:30 (27 mL), followed by a final washing step elution with CH_2_Cl_2_/CH_3_OH 90:10 (9 mL). Both organic fractions were dried with evaporator, transferred in a vial with MeOH and finally dissolved in 200 µl LC/MS grade MeOH to be analyzed (injection volume 20 μl). Experiments were performed in duplicate. A standard calibration curve was obtained by increasing concentration of C17-Sph and C17-S1P from 5 to 500 ng/mL.

### LC/MS analysis

LC-MS analysis was performed on Q-Exactive Orbitrap in positive ion mode,using for the chromatographic separation a Luna-RP column 5 μm (150 × 2 mm, Phenomenex, Italy) in agreement to Schmidt *et al*.^24^. MS source parameters were as follows: electrospray voltage 5.4 kV, capillary temperature 400 °C, s-lens rf level 45, auxiliary gas flow rate 30, sheath gas flow rate 30. Full MS scans were acquired within 200–500 with a mass resolution of 70,000. The target value (AGC) was 1e6 and the maximum allowed accumulation time (IT) was 100 ms. SpH and S1P ions (*m/z* 300.2892 and 380.2556 for [M + H]^+^ respectively) were selected and fragment with energy of 25 eV. Diagnostic fragments at *m/z* 282.28 and 252.27 were observed for SpH and at *m/z* 264.27 for S1P^37,38^.

### Statistical analysis

Statistical analysis was performed using Graphpad Prism software version 6.0 (Graphpad Software Inc., San Diego, CA, USA). Data were compared with unpaired Two-tailed t-test, one-way ANOVA statistical test followed by Tukey’s test, Mann-Whitney non parametric test. p values less than 0.05 were considered statistically significant.

## Electronic supplementary material

Supplementary Data
