# Supplementary material for: Sphingosine Kinases promote IL-17 expression in human T lymphocytes
Source: Sci Rep. 2018 Sep 5;8:13233. doi: 10.1038/s41598-018-31666-1 (PMC6125344; doi:10.1038/s41598-018-31666-1)
Supplement: Supplementary file 1 — Supplementary Data [file 41598_2018_31666_MOESM1_ESM.docx]

**SUPPLEMENTARY INFORMATIONS**

**Sphingosine Kinases promote IL-17 expression in human T lymphocytes.**

Giusi Barra^1^, Alessio Lepore^4^, Miriam Gagliardi^2^, Domenico Somma^4^, Maria Rosaria Matarazzo^2^, Francesca Costabile^1^, Giuseppe Pasquale^1^, Alessio Mazzoni^3^, Francesco Annunziato^3^, Carmela Gallo^6^, Genoveffa Nuzzo^6^, Angelo Fontana^6^, Antonio Leonardi^4^ and Raffaele De Palma^1,5*^

**Supplementary table S1: primers sequences**

| primer | sequence |
| --- | --- |
| sphk1 forward | 5’- ggttatggatccagcgggcg -3’ |
| sphk1 reverse | 5’- ttccgccgctcagtgagca-3’ |
| sphk2 forward | 5’- atctctgaagctgggctgtcc-3’ |
| sphk2 reverse | 5’- ctcccagtcagggcgatcta-3’ |
| il-17a forward | 5'-cttggaatctccaccgcaat-3' |
| il-17a reverse | 5'-cacgttcccatcagcgttg-3' |
| il-17f forward | 5’- cgcgtttccatgtcacgtaa-3’ |
| il-17f reverse | 5’-gcctgtacaacttccgaggg-3’ |
| il-22 forward | 5'-gctggctaaggaggctagctt-3' |
| il-22 reverse | 5'-tcagatagcagcgctcactca-3' |
| stat 3 forward | 5’- catgctgaccaacaatccca-3’ |
| stat 3 reverse | 5’-tggaggagaactgccagctc-3’ |
| rorc2 forward | 5’-tgctgagaaggacagggagccaa-3’ |
| rorc2 reverse | 5’-ggaagaagcccttgcacccctc-3’ |
| 18s forward | 5’- ggcgacgacccattcgaac-3’ |
| 18s reverse | 5’-aggcacggcgactaccatc-3’ |





**Supplementary figure 1: Sphingosine kinases expression in T lymphocytes**

**Supplementary figure 1A) Western blotting analysis of human SphK1 and SphK2 production in Th17 clones and CD4+ T cells.**

Western blotting analysis was performed on lysates obtained from Th17 cell clones and TCD4+ lymphocytes to evaluate Sphk1 and Sphk2 protein levels. Antibodies against Sphk1 and Sphk2 were used. B actin 1 was included as a loading control.

**Supplementary figure 1B) mRNA expression of Th17 markers**

IL-17F, IL-22, RORC2 and STAT 3 mRNA expression by CD4+ T cells (ctr) and polarized Th17 cell was evaluated by real-time quantitative PCR after12 days of culture. Results were normalized to 18S mRNA and analyzed by ΔΔCt method. Values on y-axis represent fold change in mRNA levels compared to control. Data indicate mean ± s.d. obtained from six separate experiments performed in triplicate. Unpaired Two-tailed t-test was used for statistical analysis. Asterisks (*) represent statistical significance of: ** p< 0,001 *** p < 0,0001.

**Supplementary figure 1C)** **SphK1, SphK2 mRNA expression by Th1 and Th2 lymphocytes polarized in vitro.**

Sphks expression levels were analyzed through quantitative Real Time PCR. Results were normalized to 18S mRNA and analyzed by ΔΔCt method. Values on y-axis represent fold change in mRNA levels compared to control, represented by TCD4+ lymphocytes cultured in non polarizing condition. Data indicate mean ± s.d. obtained from six separate experiments performed in triplicate. Unpaired Two-tailed t-test was used for statistical analysis (*p< 0,01, **p< 0.001).





**Supplementary figure 2: Sphingosine kinases expression transfection efficciency**

**Supplementary figure 2A) Transfection efficiency and cells viability**

Representative flow cytometric analysis of GFP expression by TCD4+ lymphocytes after nucleofection with Amaxa nucleofector technology (Lonza) with 2 μg of vectors associated to GPF and containing SphK1 or SphK2 overexpressed; empty plasmid was used as control. After 48 hours of culture, cells were harvested and stained with 7AAD to test cells viability. The gate (R3) was placed on the 7AAD negative population and 5,000 of gated cells were acquired. Numbers in plots indicate percentage of gated cells positive for GFP.

**Supplementary figure 2B) Western blotting analysis of human SphK1 and SphK2 production in CD4+ T cells after nucleofection.**

Western blotting analysis was performed on lysates obtained from TCD4+ lymphocytes after nucleofection with 2 μg of vectors associated to GPF and containing SphK1 or SphK2 overexpressed; empty plasmid was used as control. After 48 hours of culture, cells were lysed and analyzed. Antibodies against Sphk1 and Sphk2 were used. B actin 1 was included as a loading control.


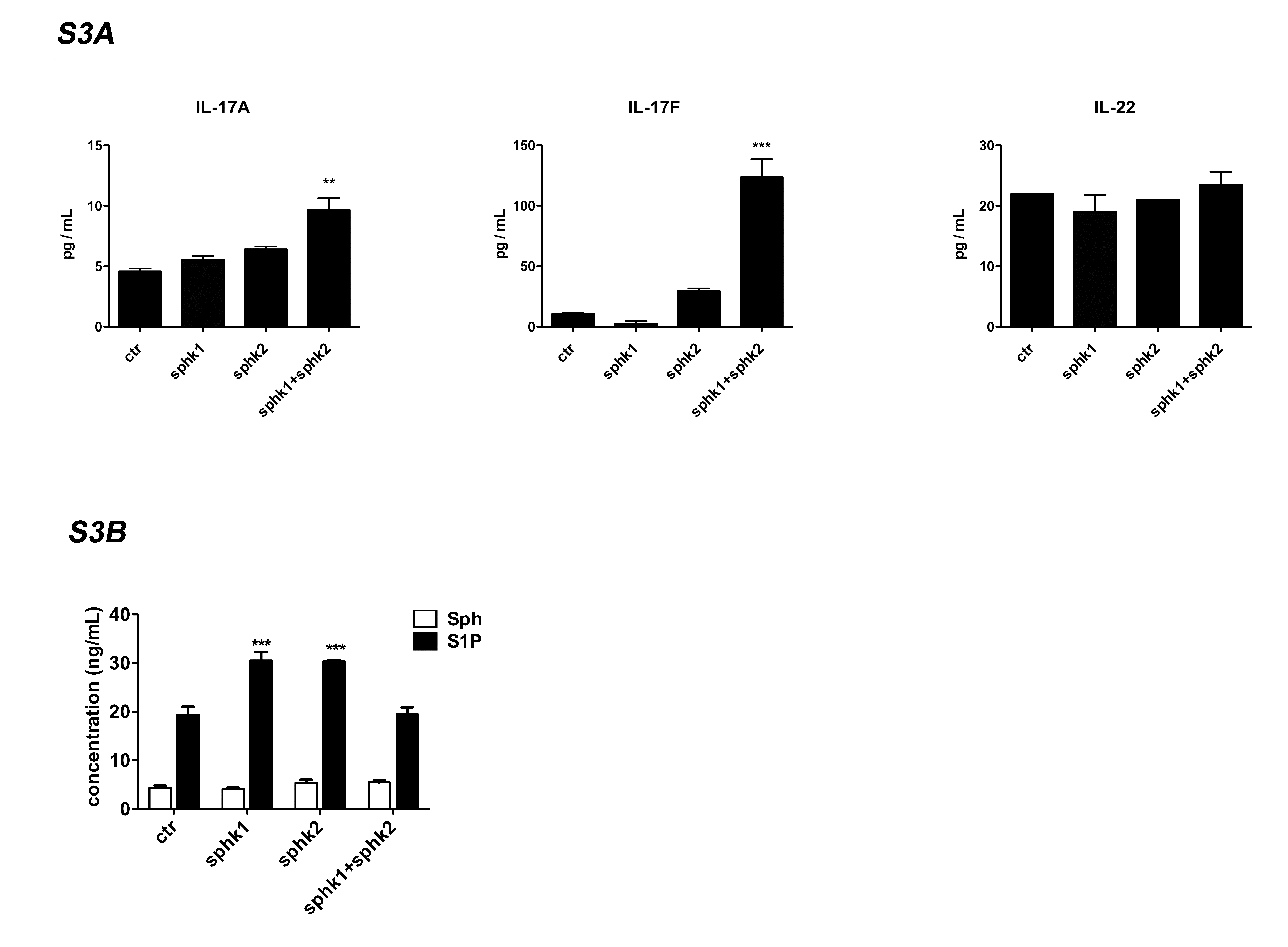


**Supplementary figure 3: Effects of sphingosine kinases overexpression**

**Supplementary figure 3A**) **Cytokines production in supernatants of TCD4+ cells after nucleofection**

Nucleofection was conducted through Amaxa nucleofector technology (Lonza) with 2 μg of vectors containing SphK1, SphK2 or SphK1+SphK2; empty plasmid was used as control. Supernatants were collected after 48 hours after transfection. Cytokines production was measured with human multiplex-cytokine kits (Millipore) using Luminex technology. Bars indicate mean ± s.d. One way ANOVA test, followed by Tukey's test were used for statistical analysis. Asterisks (*) represent statistical significance of: ** p< 0,001.

**Supplementary figure 3B) Sph and S1P production in TCD4+ supernatants after nucleofection**

Level (ng/mL) of S1P and Sph in supernatants of CD4+ were measured 48hours after nucleofection of ctr (empty plasmid), SphK1, SphK2 and both kinases. The internal standards (C17-Sph and C17-S1P) were added to the supernatants, which were processed by HRX-SPE (Chromabond ®); The recovered fractions enriched in S1P and Sph were analyzed by LC-MS-MSMS. Bars indicate mean ± s.d. One-way ANOVA test, followed by Tukey's test were used for statistical analysis (*** p< 0,001).
